# Expansion of Immature Neutrophils During SIV Infection Is Associated With Their Capacity to Modulate T-Cell Function

**DOI:** 10.3389/fimmu.2022.781356

**Published:** 2022-02-03

**Authors:** Julien Lemaitre, Delphine Desjardins, Anne-Sophie Gallouët, Mario Gomez-Pacheco, Christine Bourgeois, Benoit Favier, Asier Sáez-Cirión, Roger Le Grand, Olivier Lambotte

**Affiliations:** ^1^ Université Paris-Saclay, Inserm, CEA, Center for Immunology of Viral and Autoimmune, Hematological and Bacterial diseases (IMVA-HB/IDMIT), Paris, France; ^2^ Assistance Publique - Hôpitaux de Paris, Université Paris Saclay, Hôpital Bicêtre, Service de Médecine Interne et Immunologie Clinique, Paris, France; ^3^ Institut Pasteur, Unité HIV inflammation and persistance, Paris, France

**Keywords:** AIDS, antiretroviral treatment, neutrophils, immunomodulation, non-human primate, HIV, SIV, T cell modulation

## Abstract

In spite of the efficacy of combinational antiretroviral treatment (cART), HIV-1 persists in the host and infection is associated with chronic inflammation, leading to an increased risk of comorbidities, such as cardiovascular diseases, neurocognitive disorders, and cancer. Myeloid cells, mainly monocytes and macrophages, have been shown to be involved in the immune activation observed in HIV-1 infection. However, less attention has been paid to neutrophils, the most abundant circulating myeloid cell, even though neutrophils are strongly involved in tissue damage and inflammation in several chronic diseases, in particular, autoimmune diseases. Herein, we performed a longitudinal characterization of neutrophil phenotype and we evaluated the interplay between neutrophils and T cells in the model of pathogenic SIVmac251 experimental infection of cynomolgus macaques. We report that circulating granulocytes consists mainly of immature CD10- neutrophils exhibiting a prime phenotype during primary and chronic infection. We found that neutrophil priming correlates with CD8^+^ T cell activation. Moreover, we provide the evidence that neutrophils are capable of modulating CD4^+^ and CD8^+^ T-cell proliferation and IFN-γ production in different ways depending on the time of infection. Thus, our study emphasizes the role of primed immature neutrophils in the modulation of T-cell responses in SIV infection.

## Introduction

In the absence of a cure for HIV infection, lifelong antiretroviral treatment is necessary to control replication of the virus because of the inability of combinational antiretroviral treatment (cART) and host defenses to eradicate it. cART has increased life expectancy and the quality of life of HIV-infected patients, but is still far from being a cure (1, 2). Despite cART, non-AIDS related co-morbidities (metabolic, neurodegenerative, and cardiovascular diseases) normally associated with aging have been shown to be more frequent and occur earlier in the HIV-infected than aged-matched general population (3). Such an increased frequency of co-morbidities was suggested to contribute to the persistence of chronic inflammation (4). Even under long-term cART, HIV-infected patients have a higher level of soluble inflammatory (ex. IL-6) and gut epithelial dysfunction markers (ex. I-FABP) (5).

Nevertheless, the exact mechanisms and cells involved in the persistence of low-grade chronic inflammation in cART patients are still not clear. Residual HIV replication and microbial translocation potently activate monocytes, dendritic cells, and macrophages, leading to chronic inflammation (6–8). However, one of the most potent pro-inflammatory myeloid cells subset, neutrophils, have been overlooked in HIV-infection (9). In spite of being considered as short-lived phagocytic cells, new studies have demonstrated an increased neutrophil life expectancy in certain conditions and diseases, reaching 5.4 days, with a capacity to exert immunomodulatory functions (10–13). Neutrophils have been shown to be involved in the pathogenesis of metabolic, cardiovascular, and neurological diseases, which are also non–AIDS related comorbidities (14). Neutrophil infiltration in the gut has been recently shown in HIV-infected patients under cART and has been reported to be associated with colonic epithelial cell apoptosis and microbial translocation in SIV-infected macaques (15–17). Neutrophil survival in the gut has been shown to be increased in chronic HIV infection, a feature that is associated with the pro-inflammatory function in inflammatory bowel disease (18, 19).

Circulating neutrophils with increased spontaneous reactive oxygen production (ROS), CD62L shedding, and CD11b upregulation have been described in chronically HIV-infected patients (20, 21). This mechanism, called priming, refers to a process in which neutrophil exposure to inflammatory mediators increases responsiveness to subsequent agonist stimuli (22, 23). First shown by increased ROS production prior to any stimulation, priming also affects neutrophil longevity, phenotype, shape, and deformability. This mechanism, driven by soluble factors (cytokines, microbial products, and chemo-attractants), relies on assembly of the NAPDH complex and is also accompanied by granule release, phenotypic changes, cytokine production, transmigration, and delayed apoptosis (23). Thus, depending on the inflammatory environment, primed neutrophils are armed to respond more efficiently against invasive pathogens.

Neutrophils have long been considered to be a homogeneous population but there is increasing evidence of neutrophil diversification under inflammatory conditions (11, 24). Neutrophil subpopulations can be distinguished by physical, phenotypic, and functional changes that can have a beneficial or detrimental impact on disease (24). Only a few studies have reported neutrophil diversification in HIV/SIV infection (21, 25–27). During chronic HIV infection, a subset of neutrophils expressing PD-L1 were shown to suppress specific T cell-mediated IFN-γ production *via* PD-1/PD-L1 and ROS-dependent mechanisms (25).

We previously observed neutrophil diversification in chronically SIV infected macaques with evidence of increase frequency of immature neutrophils (28). In this study, we hypothesized that neutrophil diversification occurs early in SIV infection and is associated with changes of bone marrow neutrophil. We used flow cytometry approach to investigate neutrophil dynamic in non-human primates (NHPs) infected with pathogenic SIVmac251 during the primary and chronic stages of infection, and also, in ART controlled SIV infection. We report that circulating neutrophils in primary and chronic SIV infection are mostly comprised of primed (CD62L^low^) and immature (CD10^-^) neutrophils. Associated with neutrophils diversification, we also previously identified an impairment of Gram-negative bacteria phagocytosis by neutrophil, but less is known about their capacities to modulate T cells responses (28). We further hypothesized that neutrophil diversification is associated with immunomodulatory functions against T cells. This prime immature phenotype was associated with the capacity of neutrophils to modulate T-cells proliferation and cytokines production. Thus, our results suggest the involvement of primed immature neutrophils in the modulation of T-cell response in SIV infection.

## Materials and Methods

### Nonhuman Primates and Sampling

Twelve cynomolgus macaques (*Macaca fascicularis*), imported from Mauritius, were housed at the IDMIT animal facility at the CEA (Fontenay-aux-Roses, France). Animals expressing the H6 MHC haplotype were excluded because of their natural capacity to spontaneously control SIVmac251 viremia and disease progression (29). All animals were infected by intravenous inoculation with 1,000 times the animal infectious dose 50% (AID50) of a pathogenic SIVmac251 isolate and followed for more than 12 months (30). Six SIV-infected animals (Group 1) were subcutaneously treated daily, starting from 28 days post infection (dpi), with a combination of two nucleoside reverse-transcriptase inhibitors, emtricitabine (40 mg/kg) and tenofovir disoproxil fumarate (5.1 mg/kg), and one integrase inhibitor, dolutegravir (2.5 mg/kg) (31). The six remaining animals (Group 2) were treated by the same route and posology but at month 6 of the infection. Following SIV infection, two animals reached humane euthanasia endpoint in Group 1. The first one animal was euthanized at 48 dpi for weight loss and diarrhea, and the second was euthanized at 527 dpi for weight loss and peripheral neuropathy.

Blood was drawn and bone marrow aspirates collected under anesthesia, which consisted of an intramuscular injection of 10 mg/kg zolazepam/tiletamine (Virbac, Carros, France). Blood was collected into lithium-heparin tubes (Vacutainer BD, USA) and bone marrow aspirates from the iliac crest or humeral head using a 10-ml 18G-syringe containing citrate -dextrose (10 mg/ml).

The macaque study was approved by the « Ministère de l’Education Nationale, de l’Enseignement Supérieur et de la Recherche » (France) and the ethics committee « Comité d’éthique en expérimentation animale n°44 » under reference APAFIS#2453-2015102713323361; Animals were handled in accordance with national regulations (CEA accreditation number D92-032-02) and European Directives (2010/63, recommendation N°9) and in compliance with the Standards for Human Care of the Office for Laboratory Animal Welfare (OLAW, USA) under OLAW Assurance number #A5826-01.

### Reagent and Cell Preparation for Flow Cytometry

Briefly, heparinized whole blood and citrated bone marrow were processed within 1 h following blood draw to preserve neutrophils. Phenotypic analysis were performed with two different panels based on the same core panel but markers were adapted to priming or maturation status of neutrophils. Viability staining was first performed for 15 min at room temperature, according to the manufacturer’s protocol (Live Dead, Thermofisher). Then, antibody staining was performed with the following antibodies for 15 min at room temperature: Core panel: CD11b (ICRF144, BD Bioscience), CD45 (D058-1283, BD Bioscience) CD3 (SP34.2, BD Bioscience), CD8a (RPAT8, BD Bioscience), HLADR (L234, Biolegend), and CD66abce (TET2, Myltenyi Biotec), CD20 (2H7, BD Bioscience); CD32a (FLI8.26, BD Bioscience), CD14 (M5E2, BD Bioscience), CD16 (3G8, BD Bioscience), CDw125 (A14, BD Bioscience); Priming panel: CD64 (10.1, BD Bioscience), CD62L (SK11, BD Bioscience), CD89 (A59, Biolegend); Maturation panel: CD49d (9F10, Biolegend), CD10 (HI10a, Biolegend), CD101 (BB27, Biolegend). For MDSC analysis, PBMC were stained using the same protocol and the same core panel and following additional antibodies were added: CD10 (HI10a, Biolegend), CD101 (BB27, Biolegend), CD33 (AC104 3E3, Miltenyi Biotec).

Samples were then lysed and fixed using BD FACS Lysing (BD Bioscience) for 15 min. After an additional wash with PBS, acquisition was performed on a BD FORTESSA flow cytometer (BD Bioscience) and analyzed using FlowJo software. Gating strategies are detailed in **Supplementary Figures S1**, **S2**.

### Cell Isolation

Mononuclear cells and granulocytes were isolated by density gradient centrifugation (Ficoll-Paque; GE Healthcare Life Sciences) of blood. Using this technique, we obtained 95.6% (± 3) neutrophil purity in the globular fraction. The isolation procedure was further shown to not induce PMN activation based on CD62L and CD11b expression.

### Neutrophil Cells Sorting and Cytology

For cell sorting by flow cytometry, whole blood or bone marrow from two uninfected animals were first lysed using NH_4_Cl, then, FcRs were blocked using cynomolgus macaque serum. Cells were counted and incubated for 30 min with the following antibodies: CD11b (ICRF44), CD45 (D058-1283), CDw125 (REA705), CD123 (7G3), CD3 (REA994), CD20 (LT20), CD8a (BW135/80), CD16 (REA423), CD10 (HI10a), CD14 (TUK4) CD32a (IV.3), and CD66 (TET2). Cell sorting was performed using a FACSAria I flow cytometer (Becton Dickinson). The sorted population was smeared with a cytocentrifuge (Cytospin 2, Thermo Scientific) and then stained using May-Grünwald Giemsa. Images were acquired using a Nikon Eclipse 80i with Dxm 1200C digital camera at 60x magnification. Cells were identified by morphological criteria by a cytologist. Promyelocytes and myelocytes were considered to be pre-neutrophils (PreN), metamyelocytes and band cells immature neutrophils, and segmented neutrophils mature cells.

### Neutrophil/PBMC Co-Culture for Cytokine and Proliferation Assay

TCR-stimulated (Cytostim NHP, Miltenyi) T-cells were cultured with or without autologous PMNs at a 1:1 or 5:1 PMN to PBMC ratio (**Supplementary Figure S3**). For the measurement of intracellular cytokines, brefeldin A (Sigma) was added after 1 h of stimulation to block protein transport to the Golgi. For the proliferation assay, PBMCs were stained with 1 µM CFSE (Cell Trace, Invitrogen) for 5 min and the reaction stopped using cold FCS. Co-cultures were maintained for 16 h for the cytokine assay and 96 h for the proliferation assay at 37°C and 5% CO2 before flow cytometry analyses.

For both assays, viability staining (Live Dead, Thermofisher) was performed at 4°C for 20 min, and for the cytokine assay, cells were permeabilized and fixed (Cytofix/cytoperm, BD Biosecience). Then, cell staining was performed for 30 min at 4°C with the following antibodies: IFN-γ (B27), TNF-α (Mab11), IL-2 (MQ1 17H12), CD4 (L200), CD8 (RPA-T8), CD69 (FN50), IL-17A (N46-653), and CD3 (SP34-2).

### Statistical Analysis

Statistical analyses were carried out using GraphPad Prism 8 software (GraphPad Software Inc.). We used the Friedman test with Dunn’s multiple comparison for statistical analyses of multiple timepoints from the same animal. A Mann-Whitney non-parametric test was used to compare data from Group 1 and Group 2. We performed a Wilcoxon matched-pair signed-rank test to analyze the impact of PMNs on T-cell proliferation and cytokine production. In graphs, the thresholds for statistical significance are indicated as follows: *p < 0.05, **p < 0.01, ***p < 0.001.

## Results

### Neutrophil Priming in Acute and Chronic SIV Infection

**Figure 1:**
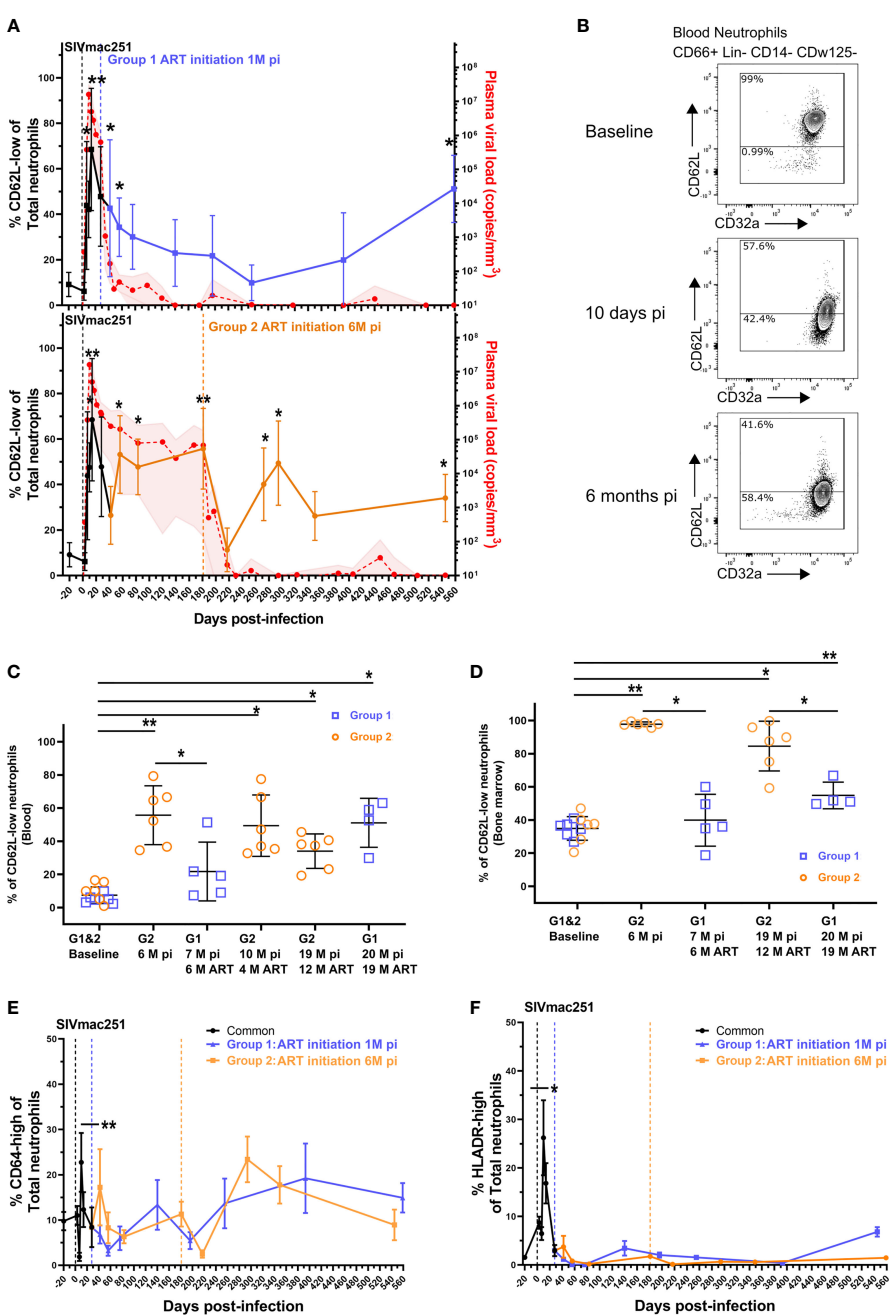
Neutrophil priming during the course of SIV infection and cART. **(A)** Follow-up of blood CD62L-low neutrophil frequency and plasma viral loads in both groups. Values shown in the graphs indicate the mean with the SD of the CD62L-low frequency and the mean, with the range, of plasma viral load. The black curve represents the data points of the 12 animals from both groups before cART initiation. *p < 0.05, **p < 0.01 by the paired Friedman test with Dunn’s multiple comparison of the CD62L-low frequency compared to baseline values for the 6 animals studied in each group. The blue (upper graph) and orange (lower graph) curves represent the data in the early cART and late cART treated animals. The red curve shows the plasma viral load for the 12 animals till 28 dpi and for the 6 animals included in each group for the latter time point. **(B)** Representative flow cytometry data plots displaying CD62L and CD32a expression in CD66+ Lin- CD14- CDw125- neutrophils from one macaque before cART. **(C, D)** Frequency of CD62L-low PMNs in blood **(C)** and bone marrow **(D)** during different stages of infection and cART. The values in the graphs indicate the mean with the SD of each neutrophil population. *p < 0.05, **p < 0.01 by the paired Friedman test with Dunn’s multiple comparison and Mann Whitney tests for unpaired comparisons. **(E, F)** Frequency of CD64-high and HLADR+ PMNs in the blood for both groups. Before cART initiation for Group 1, both groups are represented together in the black line and then separated. The values in the graphs indicate the mean with the SD of the CD64-high or HLADR^+^ PMN frequency. *p < 0.05, **p < 0.01 by the paired Friedman test with Dunn’s multiple comparison.

Neutrophil priming has been identified in chronic SIV and HIV infections (20, 21, 28) but the dynamics of neutrophils during the course of infection are still poorly defined. Because persistence of neutrophils priming could be associated with differences in functionality, we characterized these changes in SIV-infected cynomolgus macaques from acute to chronic phases and under cART. We evaluated the impact of early cART on neutrophil priming by separating the animals into two groups according to the time of treatment initiation (Group 1: cART initiated at 28 dpi, Group 2: cART initiated at 180 dpi). We characterized SSC^high^ CD45^+^ CD66^+^ Lin^-^ CD14^-^ CDw125^-^ neutrophils by multiparameter flow cytometry approach in both fresh blood and bone marrow samples (**Supplementary Figure S1A**). CD62L shedding has been previously associated with neutrophil priming in HIV-1 infection (21). We therefore considered the CD62L^low^ population as of primed neutrophils. We also used the expression of other surface markers, such as CD11b and CD64, as surrogates of priming, along with PD-L1 and HLA-DR for activation (32, 33). First, there were no significant differences in overall neutrophil blood counts between groups or timepoints (p = 0.915) (**Supplementary Figure S1B**). Nonetheless, all animals showed a significant increase in the frequency of primed CD62L^low^ neutrophils (42.9% in average of blood neutrophils (p < 0.05) at 7 dpi, reaching an average of 74.6% of blood neutrophils (p < 0.01) by 14 dpi concomitant with increase in viremia, during primary infection (**Figures 1A, B**). After primary infection, in Group 2 (cART initiated at 6 months pi) the frequency of CD62L^low^ neutrophils transiently decreased at 42 dpi (26.45%) prior a rebound at 60 dpi (53.18%), reaching to 55.7% by six months pi (p < 0.01). Thus, SIV infection induced neutrophil priming, shown by changes in the distribution between CD62L^low^ and CD62L^high^ neutrophil populations after SIV-infection, but did not affect total neutrophil blood counts.

We next evaluated the impact of cART on the CD62L^low/high^ neutrophil distribution in blood and for some time-points in BM. The initiation of cART 28 dpi (Group 1) was associated with a progressive reduction in the frequency of CD62L^low^ neutrophils, reaching basal levels after six months of cART (**Figure 1C**). Unfortunately, one animal from the Group 1 reached human endpoint criteria (weight loss >20%, persistent diarrhea and dehydration) and was euthanized at 48 dpi. After six months on ART, the frequency of primed neutrophils in Group 1 was significantly lower than that in the untreated controls after 6 months of infection (Group 2 before treatment initiation) (p < 0.05) (**Figure 1C**). In group 2, when cART was initiated at six months pi, the frequency of primed neutrophils transiently decreased after one month of treatment (**Figure 1A**) but increased thereafter, and was still significantly higher than baseline after 12 months of cART (19 months pi: baseline = 9.6% vs 12M ART = 34.1%, p < 0.05) (**Figure 1C**).

In Group 1, after 19 months under cART (20 months pi), the frequency of primed neutrophils significantly increased to 51.1% of blood neutrophils (baseline = 5.3%, p < 0.05). To note, although cART transiently restored the CD62L^low/high^ distribution when initiated early but long term alterations were observed in both groups. Similarly, the percentage of CD62L^low^ neutrophils in the bone marrow was significantly higher at 6 months pi in non-treated macaques than at baseline (baseline = 35.2%, Group 2 = 97.8%, p < 0.01) (**Figure 1D**) or than in early treated macaques (Group 1 = 39.9%, p < 0.01). The CD62L^low^ neutrophil frequencies were significantly higher in the bone marrow after long-term cART in both groups of treated animals (12 months of cART for Group 2 (p < 0.05) and 18 months of cART for Group 1 (P < 0.01)). However, early-treated animals (Group 1) still exhibited lower frequencies of CD62L^low^ neutrophil than late-treated Group 2 (p < 0.05) (**Figure 1D**).

To better characterize the activation profile of neutrophil, CD64 and HLADR surface expression were evaluated. Both markers were significantly elevated on neutrophils at the peak of viral replication (p < 0.01 and p < 0.05, respectively) and showed non-significant fluctuations during the follow-up, independently of the time of initiation of cART (**Figures 1E, F**). In addition, PDL1 expression have been shown to be elevated in PLWH, so we follow this marker expression on neutrophil and this marker did not increase during the entire follow-up period of the study (**Supplementary Figure S1C**).

These results show that significant changes in the neutrophil priming occur during pathogenic SIV infection of macaques, which are not fully corrected by cART treatment, even when cART is initiated very early (28 dpi). Nevertheless, there is a significant impact of cART on bone marrow neutrophils where CD62L expression is improved.

### SIV Infection Influences Blood Neutrophil Maturation

**Figure 2:**
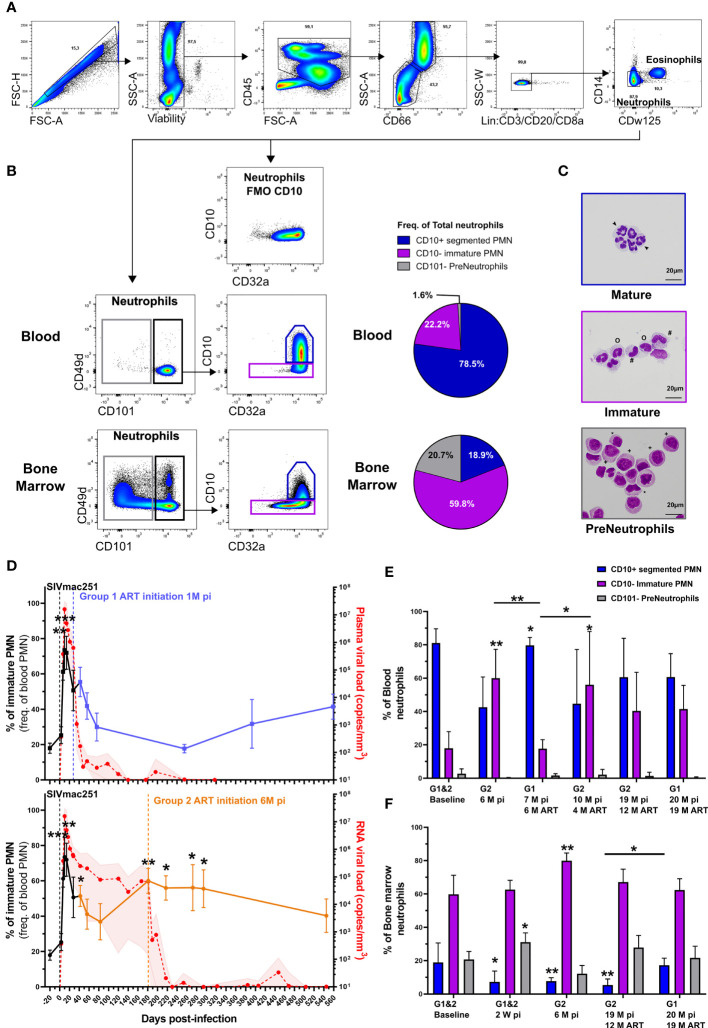
Circulation of immature neutrophils in primary and chronic SIV infection. **(A)** Gating strategy used to identify neutrophils in blood and bone marrow from cynomolgus macaques. Neutrophils were identified as CD45+ SSC-A+ CD66+ lin- CD14- and CDw125-. **(B)** Gating strategy with representative plots of different neutrophil maturations stages obtained in blood and bone marrow from a healthy cynomolgus macaque. Briefly, pre-neutrophils were gated on CD101- neutrophil population and mature CD10+ and immature CD10- neutrophils were then gated on CD101+ cells. Full minus one (FMO) staining of bone marrow for CD10 is also shown. Pie charts representing the distribution of different neutrophil maturation stages in bone marrow and blood from healthy cynomolgus macaques. **(C)** Representative cytology of corresponding cells obtained after FACS sorting on blood and bone marrow. CD101^-^ pre-neutrophils were composed of promyelocytes (**+**) and myelocytes (*). The CD101^+^ fraction was composed of immature CD10^-^ (**#**: metamyelocytes, **o**: band cells) and mature CD10^+^ (**arrow**: segmented PMNs) PMNs. **(D)** Frequency of immature PMNs in the blood during SIV infection and treatment in Group 1 and Group 2. Before cART initiation for Group 1, both groups are represented together in a black line during the first month and then separated. The values in the graphs indicate the mean with the SD for both groups, p-value indicates a significant difference to baseline, with *p < 0.05, **p < 0.01, ***p < 0.001 by the paired Friedman test with Dunn’s multiple comparison. **(E, F)** Frequency of each PMN maturation stage in the blood and bone marrow during SIV infection. Symbol * indicates a significant difference to baseline in the same group and the bar represent a significant difference between two groups, with *p < 0.05, **p <0.01, ***p < 0.001 by the paired Friedman test with Dunn’s multiple comparison and Mann Whitney tests for unpaired comparisons.

The circulation of CD62L^low^ neutrophils upon SIV infection is associated with the viremia. In other infectious diseases this phenotype has been associated with emergency granulopoiesis and the liberation of immature neutrophils from bone marrow (34). Indeed, we previously reported circulation of immature neutrophils in late stages of chronic SIV infection by mass cytometry (28). Recent publications have differentiated between maturation stages of neutrophils using CD101 and CD10 expression (35), allowing the segregation of pre-neutrophils (CD101^-^ CD10^-^) from immature (CD101^+^ CD10^-^) and segmented neutrophils (CD101^+^ CD10^+^). We showed that these markers, combined with CD32a expression, can delineate equivalent neutrophil subpopulations in the blood and bone marrow of healthy cynomolgus macaques using cell sorting by flow cytometry associated with the evaluation of cell morphology (**Figures 2A**–**C**). In bone marrow, the CD101^-^ population consisted of a mixed population showing promyelocyte and myelocyte morphology and the CD101^+^ CD10^-^ population was composed of metamyelocytes and band cells (**Figure 2C**). As low-density neutrophils have been observed in chronic inflammatory diseases, we also analyzed the kinetic of low-density neutrophils in the PBMC fraction. Nonetheless, there was no significant difference between early treated and late treated animals (**Supplementary Figures S2A–D**).

In the blood, the frequency of immature neutrophils significantly increased during the primary infection in all animals, reaching 69% of total neutrophils by 14 dpi (p < 0.001, **Figure 2D**). In the absence of cART, the frequency of immature neutrophils transiently decreased after primary infection but then rebound to reach 60% of circulating neutrophils by 6 months pi (p < 0.01, **Figure 2D** and **Supplementary Figure S2E**). Upon six months of early cART (Group 1), the frequency of immature neutrophils was significantly lower than that in untreated animals (Group 2 before cART initiation) at 6 months pi (p < 0.01, **Figure 2E**). Upon late cART (Group 2), immature PMNs represented 56.2% of blood PMNs, significantly higher than the baseline value (p < 0.05) and that of under early cART (p < 0.05) (**Figure 2E**). Early initiation of cART restored the frequency of immature PMNs relative to the delayed treatment received by Group 2. Nevertheless, the frequency of immature PMNs in both groups was not significantly different from their respective baseline values after long-term cART (19 months for Group 1 and 12 months for Group 2).

Interestingly, primary infection was associated with an enrichment of pre-neutrophils (p < 0.05) and a depletion of mature PMNs (p < 0.05) in the bone marrow of both groups, in agreement with an acute response to infection (**Figure 2F**). At 6 months pi, immature PMNs of non-treated macaques (Group 2) were significantly enriched, representing 79.9% of bone-marrow PMNs (p < 0.01) and mature PMNs were still depleted (p < 0.01) (**Figure 2F**). The initiation of early treatment (initiation at 28 dpi) prevented mature PMN depletion in the bone marrow, while the frequency of mature PMNs in late cART (Group 2) was still lower than the baseline values (p < 0.01) after one year of cART (**Figure 2F**). These results show that, similarly to PMN priming, pathogenic SIV infection induces an imbalance between mature and immature PMN in both blood and bone marrow.

### Neutrophil Priming Correlates With CD8 T-Cell Activation During SIV Infection

**Figure 3:**
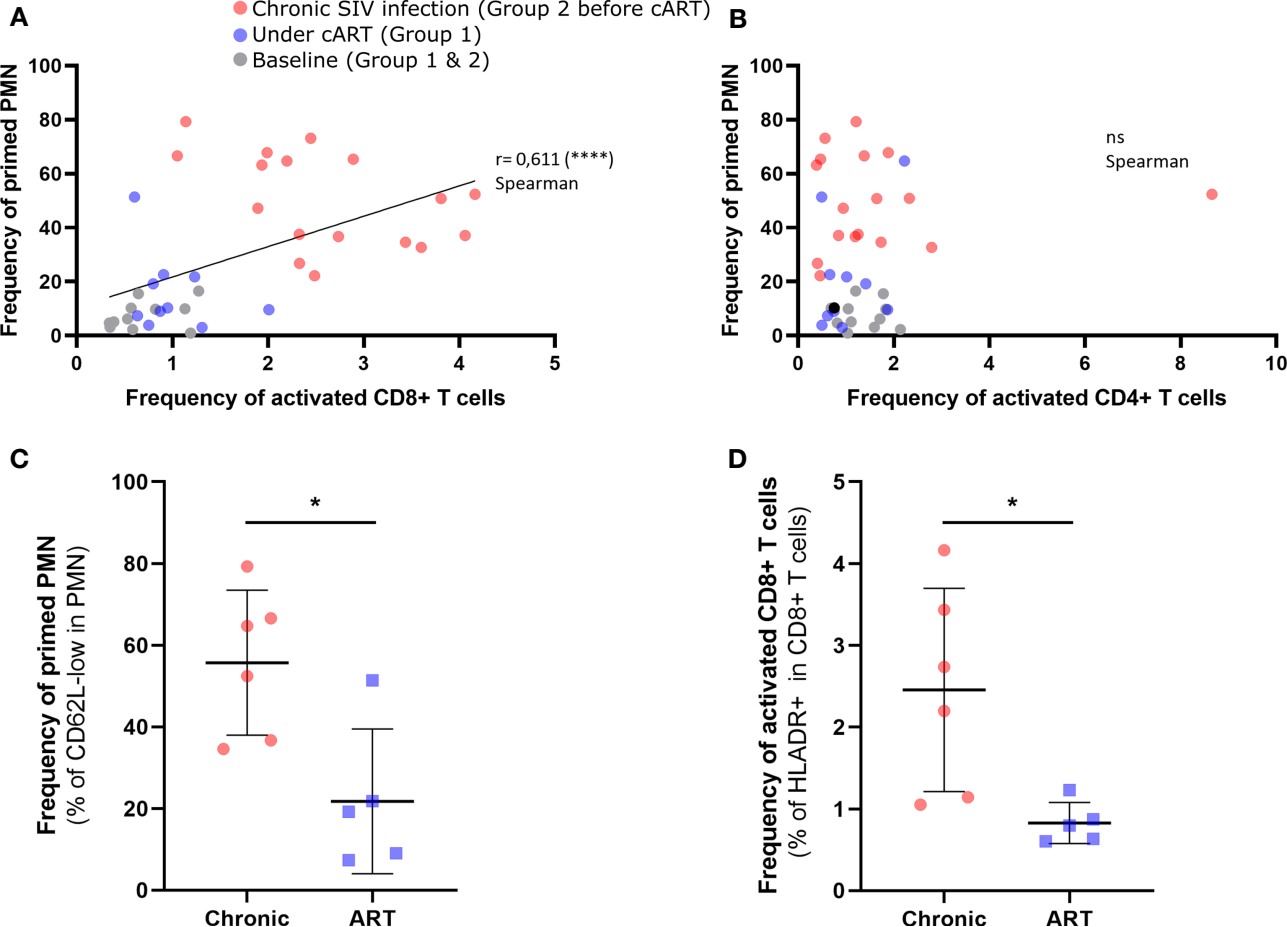
Correlation between neutrophil priming and CD4^+^ T cells and CD8^+^ T cells during SIV infection. **(A)** Correlation between the frequency of CD62L-low PMNs and that of HLADR^+^ CD8^+^ T cells (p < 0.0001). **(B)** Correlation between the frequency of CD62L-low PMNs and that of HLADR^+^ CD4^+^ T cells. The colors correspond to chronically SIV-infected animals (red; 57dpi, 3 months pi and 6 months pi), animals treated early with cART (purple; 6 months pi and 9 months pi), and baseline (gray). **(C, D)** Comparison of PMN subpopulation frequencies and activated CD8^+^ T-cell frequencies between chronically infected (6 months pi) and treated animals (7 months pi, 6 months pART). Mann Whitney test for unpaired comparisons with *p < 0.05, ****p < 0.0001. ns, non-significant.

In order to examine the potential link between neutrophil phenotype changes and immune activation, we assessed the association between the frequency of primed neutrophil and T-cells activation. As expected, flow cytometry showed a depletion of CD4+ T cells and expansion of CD8+ T cells during primary infection and early cART (Group 1) preserved CD4 T cells count during chronic phase (**Supplementary Figures S4A, B**). In addition, during the chronic phase in group 2, there was a positive correlation between viral load and activated CD8+ T cells count (**Supplementary Figure S4C**). Interestingly, the frequency of primed CD62L^low^ neutrophil correlated with CD8^+^ T-cell activation (r = 0.611, p < 0.001), but not with CD4^+^ T-cell activation, as measured by HLADR expression (**Figures 3A, B**). Nevertheless, at 42 dpi frequency of CD62L^low^ neutrophil almost correlated with CD4 T cells count (p=0.591) (S4 FigD).Viral replication plays a critical role because cART significantly reduced both neutrophil priming (p < 0.05) and CD8^+^ T-cell activation (p < 0.05) (**Figures 3C, D**). After six months of cART (Group 1, 7 months pi), there was an average of 0.8% activated CD8^+^HLADR^+^ T cells versus 2.4% in untreated macaques (Group 2 before treatment initiation, 6 months pi). This confirms that neutrophil priming occurs in association with CD8^+^ T-cell activation and suggests neutrophil priming could be a marker of immune activation in patients.

### Neutrophils Stimulate T-Cell Cytokine Production and Modulate T-Cell Proliferation in Acute and Chronic SIV Infection

As reported for several distinct diseases in humans, neutrophils can have pro-inflammatory or immunosuppressive functions, especially by modulating CD4+ and CD8+ T-cells responses (12, 36, 37). Therefore, we further explored if the functional properties of T cells may be affected by the fluctuation in the dynamics of PMNs upon SIV infection and early or late cART initiation. We evaluated T-cell immunomodulation by neutrophils in SIV infection by analyzing cytokine production and proliferation by flow cytometry of TCR-activated T cells in the presence or absence of fresh and autologous neutrophils (5:1 PBMC to neutrophil ratio) (**Supplementary Figure S3A**). PBMC to neutrophil ratio 1:5 was chosen based on the immunomodulatory properties observed in uninfected animals (**Supplementary Figures S3B–E**). In the co-culture, without any TCR-stimulation, neutrophils did not induce activation or proliferation of T cells from PBMCs.

**Figure 4:**
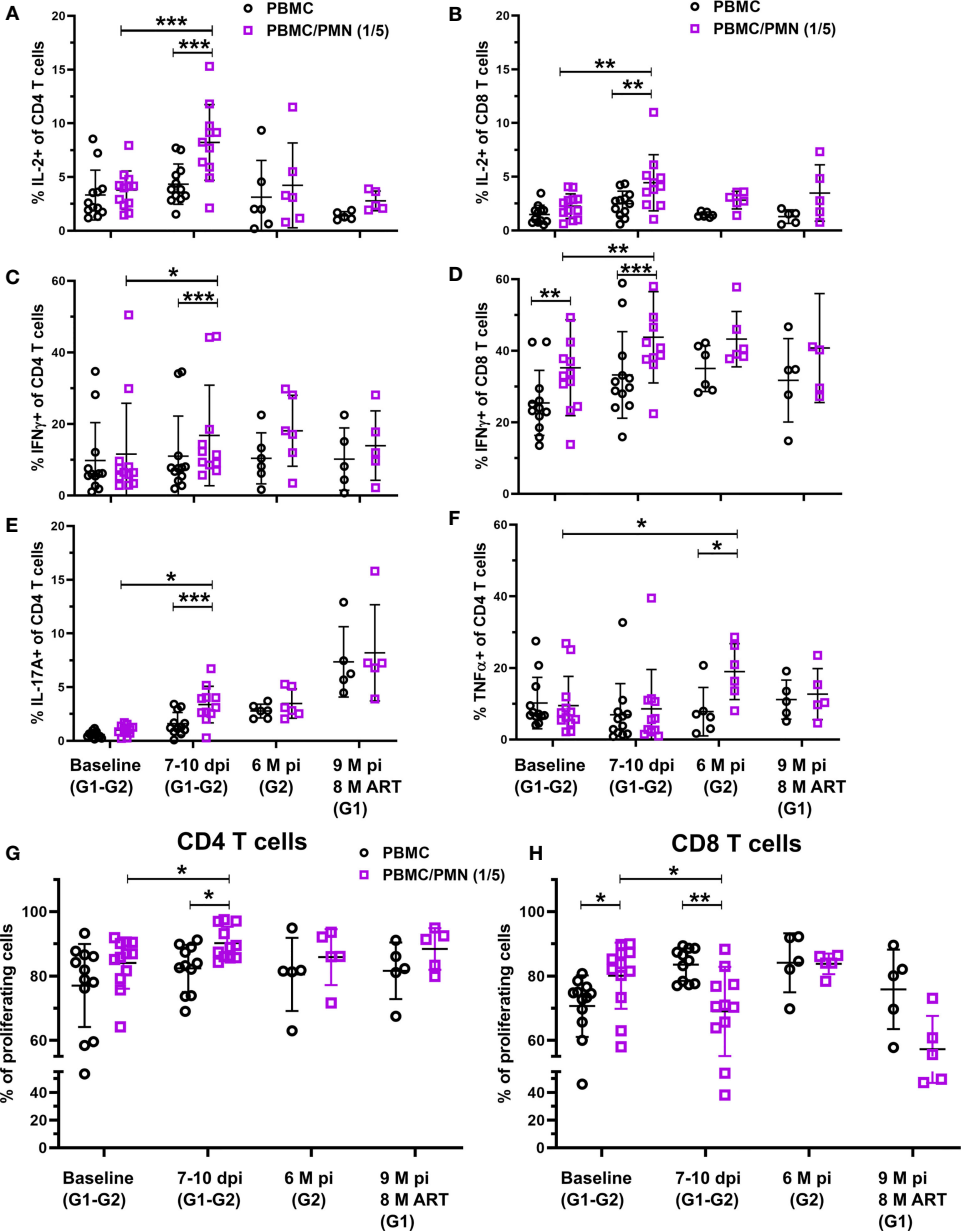
T-cell immunomodulation by neutrophils during SIV infection. **(A–F)** PBMC were cultured for 16 h with a TCR stimulation, with or without PMNs at a 5:1 PMN to T-cell ratio. T-cell cytokine production was measured by intracellular cytokine staining of IL-2, IFN-g, TNF-a, and IL-17A after co-culture. Graphs represent the frequency of CD4^+^ or CD8^+^ T cells positive for each cytokine in the absence (black) or presence of PMNs (purple). **(G, H)** PBMC were cultured for 96 h with or without PMNs at a 5:1 PMN to T-cell ratio and T cell stimulation. T-cell proliferation was measured by CFSE dilution and the populations identified by flow cytometry. Graphs represent the frequency of CD69^+^ CD4^+^ T cells and CD8^+^ T cells with CFSE dilution. *p < 0.05, **p < 0.01, ***p < 0.001 by Wilcoxon or Mann-Whitney tests depending on pairing. Baseline: Groups 1&2, Acute week 2: Groups 1&2, Chronic: Group 2 (6 M p.i.), Chronic cART: Group 1 (7 M p.i., 6M pART).

Before infection, neutrophils had no effect on the production of IL-2, IL-17A, or TNF-α by TCR-activated CD4^+^ or CD8^+^ T cells but were able to significantly increase IFN-γ production by CD8^+^ T cells (p < 0.01, **Figures 4A**–**F**). In contrast, during primary infection (days 7-10 pi), Neutrophils strongly enhance the capacity of CD4^+^ and CD8^+^ T cells to produce IL-2 and IFN-γ (remarkably, twice as much IL-2 when compare to cultures in absence of neutrophils (p < 0.001 and p < 0.01, for CD4^+^ and CD8^+^ T-cells respectively) (**Figures 4A**–**D**). Interestingly, the presence of neutrophils isolated during primary infection also enhanced by two fold the capacity of CD4+ T-cells to produce IL-17A (p < 0.001, **Figure 4E**). In the chronic phase, neutrophils did not significantly modulate IL-2, IFN-γ or IL17A production by T cells (**Figures 4A**–**E**). Instead, neutrophils from the chronic phase enhance TNF-α production by CD4+ T-cells when compared to the cells cultured in the absence of neutrophils (p < 0.05, **Figure 4F**). After three months of cART, the immunostimulatory properties of neutrophils returned to basal levels. This suggests an evolution in neutrophil immunomodulatory functions from the acute to chronic phase, with an overall stimulatory effect on CD4^+^ and CD8^+^ T-cell cytokine production.

We also measured the proliferation of T-cells in the presence or absence of neutrophils using CFSE dilution after 96 h of TCR activation. Neutrophils from acute infection, but not from baseline, the chronic phase or under cART favored CD4^+^ T cell proliferation (**Figures 4G, H**). In contrast, neutrophils from baseline favored CD8^+^ T-cell proliferation (p < 0.01) while neutrophils from acute phase had a suppressive effect on CD8^+^ T-cell proliferation (p < 0.01, **Figure 4H**). This immunosupressive effect was lost with neutrophils from non-treated animals during chronic infection (6 months pi), but was still observed in cART-treated animals after eight months of cART, although the low number of animals studied may have been insufficient to reach statistical significance (p = 0.0625). These results show that the immunomodulatory function of neutrophils evolves during the course of infection and that has a differential effect on CD4^+^ and CD8^+^ T cells.

## Discussion

The diversity of neutrophils in peripheral blood, notably associated with differing maturation and activation status, has been described in cancer and autoimmune diseases (11, 24, 38). New neutrophil functions, such as the modulation of T-cell responses have been described in these chronic inflammatory diseases. Neutrophils are also equipped with potent mediators (ROS and NETs for example) that induce tissue damage and maintain chronic inflammation (14). Chronic HIV-1 infection, even under cART, can be considered in most cases as a chronic inflammatory disease for which neutrophils have received little attention. A recent study demonstrated the link between survival of neutrophils in the gut and microbial translocation, a source of HIV-associated chronic inflammation (18). Thus, the characterization of neutrophil heterogeneity and immunomodulatory functions in HIV-1 infection would help to gain a better understanding of their roles in the maintenance of inflammation.

Here, we provide the first description of neutrophil diversity associated with T-cell immunomodulatory functions during SIV infection. We were able to distinguish between precursors and immature and mature neutrophils in the blood and bone marrow from SIV-infected macaques using the association of CD101, CD10, and CD32a expression. In primary infection, circulating neutrophils were predominantly composed of immature cells. This immature phenotype was accompanied with an increased level of HLADR, CD64, and CD62L shedding, suggesting neutrophil priming. Mitsumoto-Kaseida F. et al. previously correlated expression level of CD64 on neutrophils with CRP level in ART naïve PLWH (27). We did not observed such elevation in chronically SIV infected macaques probably because of the relative short duration of chronic phase compare to PLWH. Activation levels of CD8^+^ T cells, but not CD4+ T cells, correlated with the frequency of primed neutrophils. This might be due to the level of activation of CD8+ T cells, which is more elevated compare to CD4+ T cells in our model. Here, we show for the first time that neutrophils can differentially modulate CD4^+^ and CD8^+^ T-cell functions in primary infection in the macaque model. During primary infection, neutrophils were able to boost IL-2 and IFN-γ production by CD4^+^ and CD8^+^ T cells and IL-17A production by CD4^+^ T cells. Such immunomodulatory profile differed from the one observed at baseline: Neutrophil enriched in CD62L^hight^ cells provided positive modulation of IFNγ secretion for CD8 but not CD4 T cells. PMNs differentially modulated the capacity of T-cell proliferation in early infection by increasing CD4^+^ T-cell proliferation while decreasing it in CD8^+^ T cells.

The striking changes seen in peripheral blood neutrophil populations in primary infection partially resolved during the early chronic phase with the decrease of viremia, but the frequency of prime CD62L^low^ and immature CD10^-^ neutrophils increased in the late chronic phase. These results are consistent with bone marrow neutrophil analysis, in which primed and immature neutrophils increased during chronic SIV infection. The persistence of antigenic stimulation and inflammation may durably impair neutrophil production and phenotype. Early initiation of cART (four weeks post-infection) protected against the long-term neutrophil changes observed in untreated chronic SIV infection. Indeed, by six months under cART, the frequency of immature and primed neutrophils returned to basal levels. In contrast, the levels of immature and CD62L^low^ neutrophils did not normalize in animals treated at 24 weeks, despite undetectable viral loads in the plasma. This may be related to the persistence of chronic inflammation and the mechanisms that lead to chronic neutrophil activation, such as microbial translocation (39).

We show that neutrophils can differentially modulate CD4^+^ and CD8^+^ T cells. Primed CD62L^low^ and immature CD10^-^ neutrophils were largely predominant in the blood early during the primary infection and showed an immunostimulatory function on T cells. This could be related to a physiological function of this neutrophil subset, capable to increase the anti-viral capacity of T cells by increasing IL-2 and IFN-γ production by CD4^+^ and CD8^+^ T cells and IL-17A production by CD4^+^ T cells in the setting of gut barrier damage. The mechanisms responsible for this differential impact of neutrophil on CD4 and CD8 T cells remain elusive and will need further investigation. Intrinsic difference between CD4 and CD8 T cells to the direct neutrophil signals (different responses to cytokines, different metabolic pathways, different reactivity depending on the infection phase), but also the indirect effect of neutrophil on other immunomodulatory factors need to be evaluated. In parallel, we observed that neutrophils could have opposite effects on CD4^+^ and CD8^+^ T-cell proliferation. The negative aspect of such immunomodulatory properties, which could be largely beneficial in many viral infections, could be the increase in the frequency of proliferating activated CD4^+^ T cells, ideal “prey” for HIV. During chronic infection, alteration of neutrophils may contribute to chronic T-cell activation, as suggested by both the positive correlation between immature neutrophils and the percentage of activated CD8^+^ T cells, and the capacity of neutrophils to enhance of TNF-α production by CD4 T cells. Unfortunately, in the present study we did not evaluated the link between SIV reservoirs or residual replication in tissues and neutrophil phenotype or function.

It would be necessary to evaluate the impact of neutrophils on SIV-specific CD8^+^ T-cell function in this setting, considering that neutrophils could impair the antiviral response. PD-L1 has been reported to be expressed on neutrophils in humans and may be a way to impair the antiviral capacity of CD8^+^ T cells (25). In our study, we did not observe PD-L1 expression on neutrophils. We followed SIV infection without cART for only six months and PD-L1 expression by neutrophils may necessitate a longer time of infection to be up regulated. Under cART, neutrophils did not shown significant immunomodulatory properties against T cells, which included the absence of any impact on INFγ production by CD8^+^ T cells at baseline. We hypothesize that early cART could have a significant impact on neutrophils by reducing systemic inflammation, but exposure to ART may have a direct action on neutrophil. Indeed, various neutrophil subsets can produce several cytokines under stimulation (40). As immune cells are involved in chronic inflammation, it will be informative to evaluate the capacity of neutrophil cytokine production during the course of SIV-infection and under cART.

Acute bacterial and viral infections or LPS and G-CSF administration have been show to induce the circulation of immature neutrophils, known as a “left shift”, and neutrophil priming (12, 34, 41, 42). Primary SIV infection may also induce emergency granulopoiesis, with the mobilization of immature neutrophils from the bone marrow (34). However, persistent inflammation in chronic SIV infection has been shown to alter hematopoiesis (43, 44) and may lead to neutrophil impairment. Marini O. et al. showed opposite immunomodulatory functions of immature CD10^-^ and mature CD10^+^ neutrophils, which were immunostimulatory and immunosuppressive, respectively, in G-CSF-treated patients (12). In accordance with results of Marini et al., we identified significant neutrophil immunostimulatory function in primary and chronic SIV infection when the frequencies of immature CD10^-^ and primed CD62L-low neutrophils were significantly elevated. Confirmation that the observed immunostimulatory function is restricted to immature CD10^-^ neutrophil in SIV infection would require separate evaluation of the function of immature CD10^-^ and mature CD10^+^ neutrophils. The rarity of mature CD10^+^ neutrophils would be a major obstacle for such an experiment.

This study had other limitations. The definition of neutrophil priming relies on partial activation of NADPH-oxidase, associated with increased responsiveness to stimuli. We evaluated neutrophil priming using CD62L shedding, as it has been shown to be associated with NADPH-oxidase activation (23, 45), but we did not directly evaluate NADPH-oxidase function like ROS production. The NETosis is another neutrophil function, which was not evaluated in the present study, but its implication in chronic inflammation and T cells modulation remains to be explored. In peripheral blood, CD62L shedding is classically associated with primed hyper-segmented neutrophils, notably in acute bacterial infection (46). Here, immature neutrophils had similar dynamics as CD62L^low^ neutrophils suggesting a different pathophysiological process.

We used density gradient centrifugation to isolate neutrophils from the blood. Indeed, there are no available sorting methods to obtain neutrophils from cynomolgus macaques. This approach did not allow us to retain low-density neutrophils (LDNs), which remained in the mononuclear fraction. Nevertheless, we did not observe any significant changes in LDN frequency in the PBMC fraction of SIV-infected macaques. The impact of LDNs on T-cell function should be more investigated.

In summary, we show neutrophil diversity in SIV infection and its impact on T-cell functions. Primed CD62L^low^ and immature CD10^-^ neutrophils were elevated in primary and chronic SIV infection. This phenotype was associated with an increase in immunostimulatory function, resulting in T-cell proliferation and cytokine production. In turn, PMNs may contribute to chronic inflammation by modulating T-cell responses. But extensive functional characterization is needed in both macaque models and PLWH to better understand the role of immature neutrophils in HIV-1 infection. If certain neutrophil subsets are shown to act as pro-inflammatory cells, treatment aiming to modulate neutrophils could lead to new avenues of strategy aiming to reduce chronic inflammation in PLWH.

## Data Availability Statement

The original contributions presented in the study are included in the article/**Supplementary Material**. Further inquiries can be directed to the corresponding author.

## Ethics Statement

The animal study was reviewed and approved by Comité d’éthique en expérimentation animale n°44.

## Author Contributions

JL: designed the study, designed and performed the neutrophil phenotyping, sorting, and functional assay, analyzed the data, and wrote the paper. DD: designed and supervised the study, analyzed and discussed the results, and contributed to writing the paper article. A-SG: discussed the results and contributed to writing the paper. MG-P: performed the flow cytometry phenotyping for T-cells and LDNs, performed the neutrophil FACS sorting, and analyzed the data. CB: contributed to designing the study and discussed the results. BF: contributed to designing the study, discussed the results, and contributed to writing the paper. AS-C: conceived the project, designed the study, and discussed the results. RG: conceived the project, designed the study, discussed the results, and wrote the paper. OL: conceived the project, designed the study, discussed the results and wrote the paper. All authors contributed to the article and approved the submitted version.

## Funding

This work was supported by the “Programme Investissements d’Avenir” (PIA), managed by the ANR under reference ANR-11-INBS-0008, funding the Infectious Disease Models and Innovative Therapies (IDMIT, Fontenay-aux-Roses, France) infrastructure, and ANR-10-EQPX-02-01, funding the FlowCyTech facility (IDMIT, Fontenay-aux-Roses, France). We thank Gilead and ViiV for providing the antiretroviral drugs. Animal studies were supported by the ANRS and MSD Avenir, as part of the ANRS RHIVIERA pVISCONTI research program, and the SIVART ANRS-IDMIT CO1 research program. The funders had no role in study design, data collection and analysis, decision to publish, or preparation of the manuscript.

## Conflict of Interest

OL paid expert testimony and consultancy fees from BMS France, MSD, Astra Zeneca; consultancy fees from Incyte, expert testimony for Janssen, grant from Gilead.

The remaining authors declare that the research was conducted in the absence of any commercial or financial relationships that could be construed as a potential conflict of interest.

## Publisher’s Note

All claims expressed in this article are solely those of the authors and do not necessarily represent those of their affiliated organizations, or those of the publisher, the editors and the reviewers. Any product that may be evaluated in this article, or claim that may be made by its manufacturer, is not guaranteed or endorsed by the publisher.

## Supplementary Material

Supplementary Figure S1 Neutrophil counts and gating strategies used to identify neutrophils in cynomolgus macaques. **(A)** Gating strategy used in conventional flow cytometry to identify neutrophils in cynomolgus macaques for activation markers depicted in. **(B)** Follow-up of neutrophil blood counts in both groups of SIV-infected macaques. cART was initiated four weeks post-infection for Group 1 (N = 6) and 24 weeks post-infection for Group 2 (N = 6). The Friedman test was not significant for either group, irrespective of the timepoint or cART status **(C)** Frequency of PDL1^+^ PMNs during SIV infection in both groups.

Supplementary Figure S2 Phenotyping of neutrophil and MDSC populations in PBMC fractions. **(A)** Gating strategy used to identify low-density neutrophils (LDNs) (Lin^-^ CD11b^+^ CD66^+^ HLADR^-^ CD14^-^), PMN-MDSCs (CD33^+^ LDNs) and M-MDSCs (Lin^-^ CD11b^+^ CD66^+^ HLADR^-^ CD14^+^) in the mononuclear fraction. **(B–D)** Frequency of LDNs, PMN-MDSCs, and M-MDSCs in CD45^+^ PBMCs from Groups 1&2 (black) during primary infection and the separate groups during the chronic phase. There were no statistical differences using the paired Kruskal-Wallis test with Dunn’s multiple comparison. **(C, E, F)** Frequency of mature PMNs in blood with the plasma viral load for Group1 and Group 2.

Supplementary Figure S3 Gating strategy for co-culture assay and selection of the neutrophil to PBMC ratio. **(A)** Gating strategy used to evaluate T-cell proliferation and cytokine production after co-culture with PMNs. **(B, C)** Frequency of activated CD4^+^ and CD8^+^ T cells according to the neutrophil ratio and stimulation. **(D, E)** Frequency of proliferating T cells according to the neutrophil ratio and stimulation.

Supplementary Figure S4 T cells analyses during the course of SIV infection. **(A, B)** CD4+ and CD8+ T cells counts in blood during SIV infection in both groups **(C)** Correlation between frequency of activated CD8+ T cells and Log10 plasma viral load from 57 to 181 dpi in group 2. **(D)** Correlation between CD4 T cells count and frequency of primed PMN at 41 dpi. Spearman correlation p-value < 0,0001.
