# Supplementary figures and images for: Expansion of Immature Neutrophils During SIV Infection Is Associated With Their Capacity to Modulate T-Cell Function
Source: Front Immunol. 2022 Feb 3;13:781356. doi: 10.3389/fimmu.2022.781356 (PMC8851599; doi:10.3389/fimmu.2022.781356)

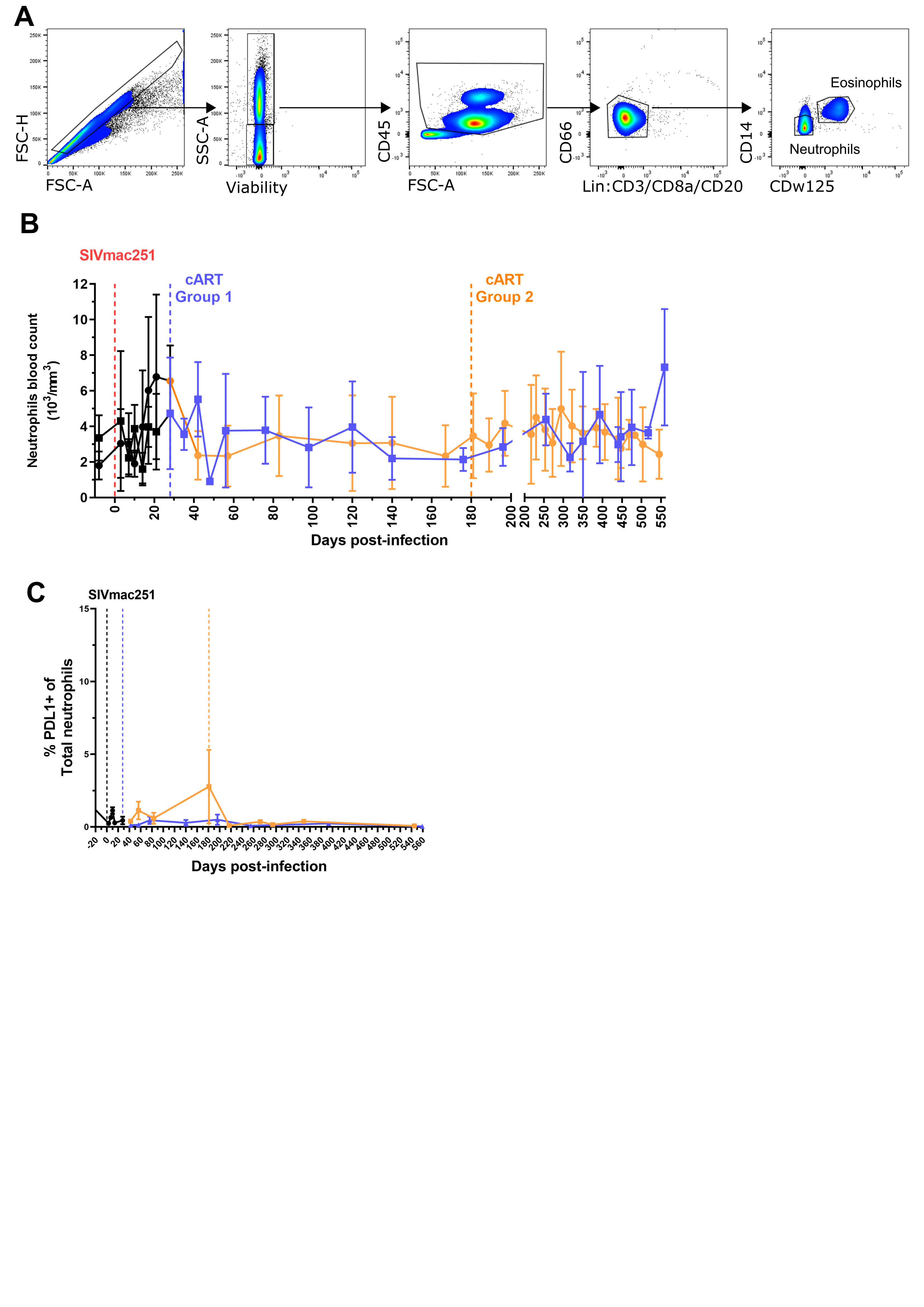

Supplement: Supplementary Figure S1 — Neutrophil counts and gating strategies used to identify neutrophils in cynomolgus macaques. (A) Gating strategy used in conventional flow cytometry to identify neutrophils in cynomolgus macaques for activation markers depicted in. (B) Follow-up of neutrophil blood counts in both groups of SIV-infected macaques. cART was initiated four weeks post-infection for Group 1 (N = 6) and 24 weeks post-infection for Group 2 (N = 6). The Friedman test was not significant for either group, irrespective of the timepoint or cART status (C) Frequency of PDL1+ PMNs during SIV infection in both groups. [file Image_1.tif]

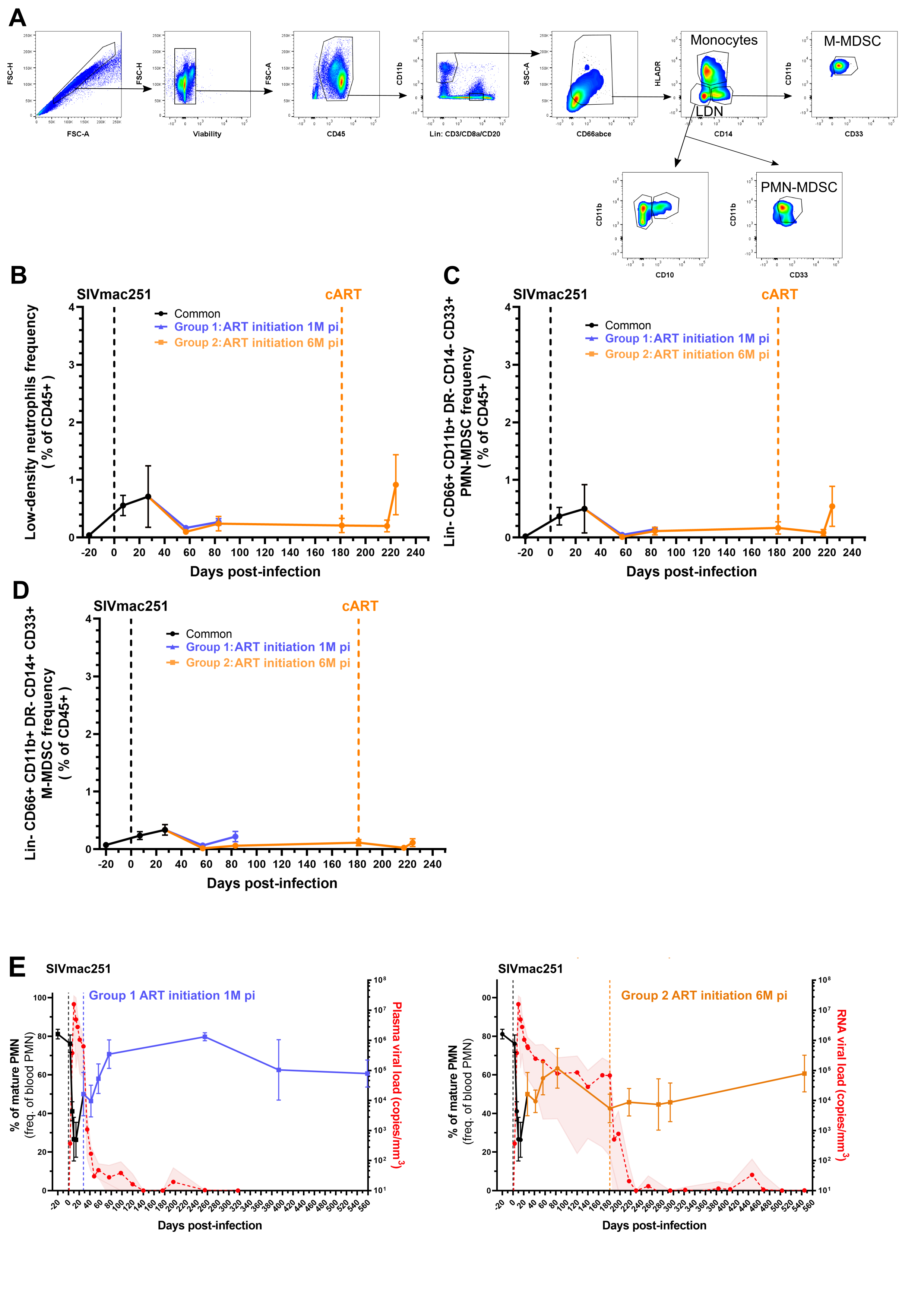

Supplement: Supplementary Figure S2 — Phenotyping of neutrophil and MDSC populations in PBMC fractions. (A) Gating strategy used to identify low-density neutrophils (LDNs) (Lin- CD11b+ CD66+ HLADR- CD14-), PMN-MDSCs (CD33+ LDNs) and M-MDSCs (Lin- CD11b+ CD66+ HLADR- CD14+) in the mononuclear fraction. (B–D) Frequency of LDNs, PMN-MDSCs, and M-MDSCs in CD45+ PBMCs from Groups 1&2 (black) during primary infection and the separate groups during the chronic phase. There were no statistical differences using the paired Kruskal-Wallis test with Dunn’s multiple comparison. (C, E, F) Frequency of mature PMNs in blood with the plasma viral load for Group1 and Group 2. [file Image_2.tif]

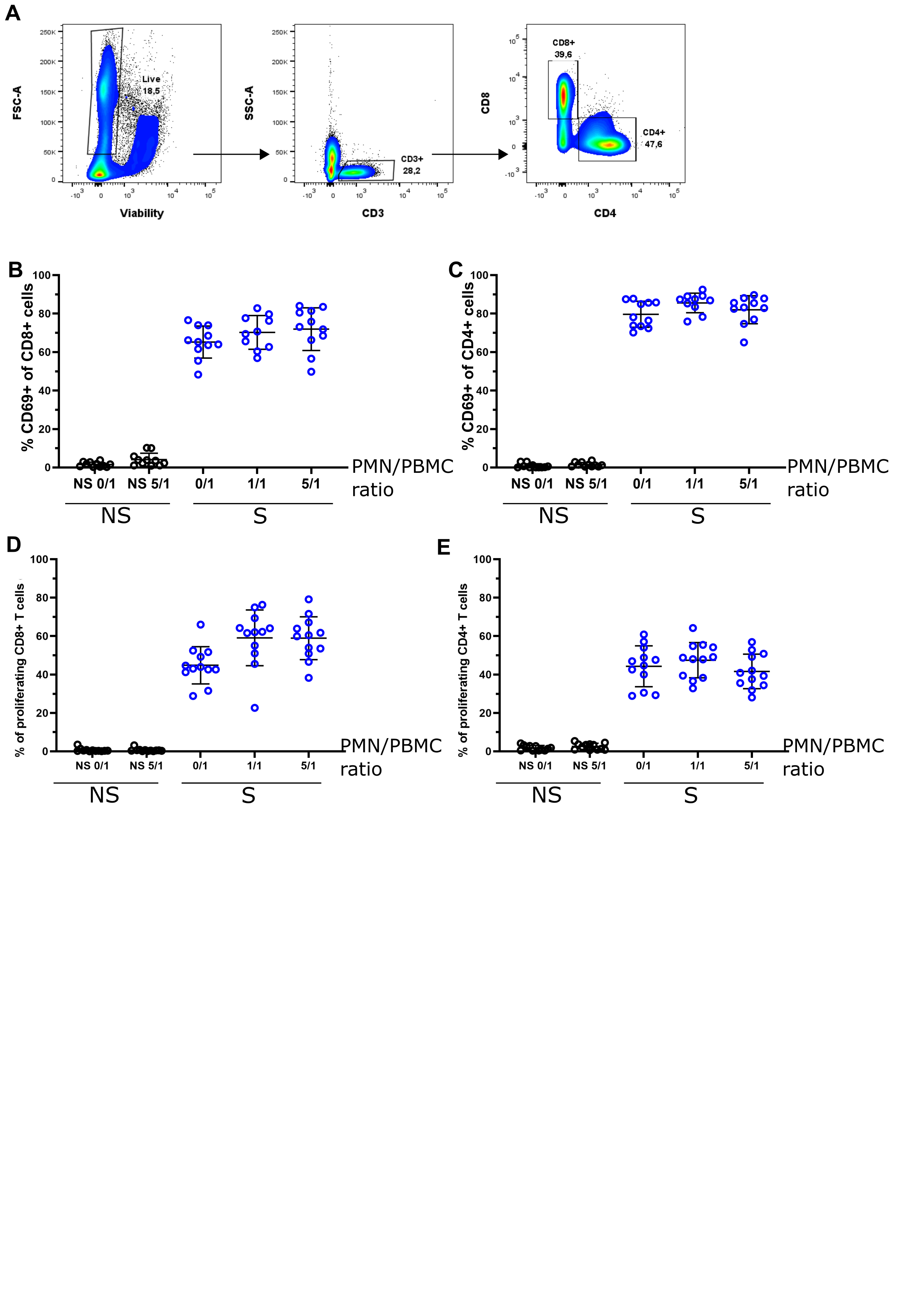

Supplement: Supplementary Figure S3 — Gating strategy for co-culture assay and selection of the neutrophil to PBMC ratio. (A) Gating strategy used to evaluate T-cell proliferation and cytokine production after co-culture with PMNs. (B, C) Frequency of activated CD4+ and CD8+ T cells according to the neutrophil ratio and stimulation. (D, E) Frequency of proliferating T cells according to the neutrophil ratio and stimulation. [file Image_3.tif]

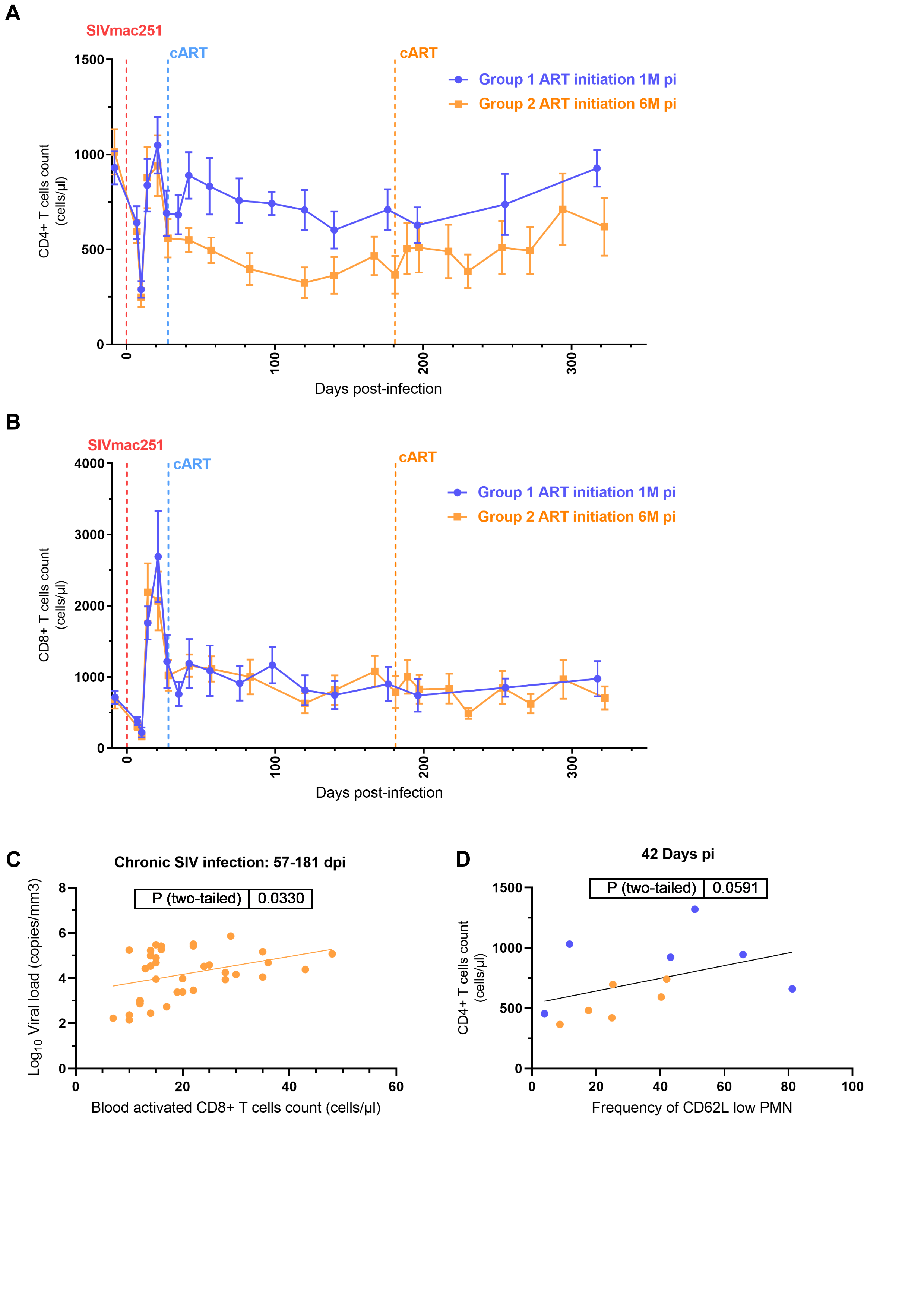

Supplement: Supplementary Figure S4 — T cells analyses during the course of SIV infection. (A, B) CD4+ and CD8+ T cells counts in blood during SIV infection in both groups (C) Correlation between frequency of activated CD8+ T cells and Log10 plasma viral load from 57 to 181 dpi in group 2. (D) Correlation between CD4 T cells count and frequency of primed PMN at 41 dpi. Spearman correlation p-value < 0,0001. [file Image_4.tif]
